# How weather instead of urbanity measures affects song trait variability in three European passerine bird species

**DOI:** 10.1002/ece3.3032

**Published:** 2017-05-28

**Authors:** Julia E. Schäfer, Marcel M. Janocha, Sebastian Klaus, Dieter Thomas Tietze

**Affiliations:** ^1^ Department of Ecology and EvolutionInstitute of Ecology, Evolution and DiversityGoethe UniversityFrankfurt am MainGermany; ^2^ Institute of Pharmacy and Molecular Biotechnology and Heidelberg Center for the EnvironmentHeidelberg UniversityHeidelbergGermany

**Keywords:** air pressure, bioacoustics, humidity, noise, soil temperature, temperature, urbanity gradient, urbanization

## Abstract

Previous studies detected an influence of urban characteristics on song traits in passerine birds, that is, song adjustments to ambient noise in urban areas. Several studies already described the effect of weather conditions on the behavior of birds, but not the effect on song traits. We investigate, if song trait variability changes along a continuous urbanity gradient in Frankfurt am Main, Germany. We examined, for the first time on a larger scale, the influence of weather on song parameters. We made song recordings of three common passerine species: the blue and great tit (*Cyanistes caeruleus* (Linnaeus, 1758) and *Parus major* Linnaeus, 1758) and the European blackbird (*Turdus merula* Linnaeus, 1758). We measured different song traits and performed statistical analyses and modeling on a variety of variables—among them urbanity and weather parameters. Remarkably, we found only few cases of a significant influence of urbanity parameters on song traits. The influence of weather parameters (air pressure, atmospheric humidity, air and soil temperatures) on song traits was highly significant. Birds in Frankfurt face high noise pollution and might show different adaptations to high noise levels. The song trait variability of the investigated species is affected more by weather conditions than by urban characteristics in Frankfurt. However, the three species react differently to specific weather parameters. Smaller species seem to be more affected by weather than larger species.

## 1 INTRODUCTION

Singing represents an exceptionally important aspect of communication in songbirds (Passeriformes). It serves to define the territory and to defend against conspecifics or other intruders as well as to attract and court females. The offspring usually learns the song from its social father (Waser & Marler, 1977). Thus, singing plays an essential role in the life cycle of songbirds.

In the contexts mentioned above, it is important that the song is transmitted and received with its whole information content to be understood by its receiver (Wiley & Richards, 1983). There are different biotic and abiotic factors, which may interfere in sound transmission. A well‐studied abiotic factor is ambient noise (Brumm, 2004; Cardoso & Atwell, 2011; Hu & Cardoso, 2010).

With increasing urbanization, anthropogenic noise intensifies. Ambient noise covers mostly lower frequencies and consequently threatens especially low‐pitched birdsong with its masking effect (Gil & Brumm, 2014). It has been shown that birds increase their amplitude and minimum frequency within a verse to avoid the masking effect of noise (Brumm, 2004; Slabbekoorn & Peet, 2003).

In this study, we use the expression “urbanity” according to Ziege et al. (2015) to describe the degree of urbanization as a quantitative measure of anthropogenic impact. In comparison with rural sites, urban areas are defined by high building density, more roads, and low vegetation cover. These differing conditions between rural and urban sites were shown to influence behavior, morphology, and other traits of birds. Studies examined the correlation between the degree of urbanity and avian fitness as measured by morphology (Bókony, Seress, Nagy, Lendvai, & Liker, 2012) and productivity (Chamberlain et al., 2009). Whereas there was no effect on morphology in house sparrows (*Passer domesticus* Linnaeus, 1758), there was a significant effect of urbanization on productivity. Productivity per nesting attempt was lower in urban areas although annual productivity was in some cases higher in cities.

Warren, Katti, Ermann, and Brazel (2006) also discussed that urbanization has greater influence on song parameters than ambient noise. This might for example be due to high buildings with many reflective surfaces which might modulate sound transmission. Therefore, to infer human influence on bird song, it is important to consider the degree of urbanization in addition to ambient noise alone (Bókony et al., 2012; Giraudeau et al., 2014; Seress, Lipovits, Bókony, & Czúni, 2014; Ziege et al., 2015).

Studies have also examined the effect of weather conditions on the behavior of birds. Passerines sing earlier with rising temperatures in spring, but later when it is rainy or cloudy (Bruni, Mennill, & Foote, 2014). Cresswell and McCleery (2003) found that the great tit adjusts its breeding biology by means of clutch size and incubation time to the weather conditions because it directly influences food supply. They alter their breeding behavior to ensure that there will be enough food when the offspring needs to be fed the most. Chase, Nur, and Geupel (2005) discussed the highly significant correlation of reproductive success with weather. Additionally, Botero, Boogert, Vehrencamp, and Lovette (2009) found that mockingbirds (family Mimidae) sing a more elaborate song if they are exposed to frequently alternating weather conditions.

We conducted this study to determine whether the effect of urbanity detected in previous studies could be reproduced in the city of Frankfurt am Main (50°7′N, 8°38′E) and to analyze the effect of weather on song parameters. Therefore, we made song recordings from three common passerines, the blue tit (*Cyanistes caeruleus* (Linnaeus, 1758)), the great tit (*Parus major* Linnaeus, 1758), and the European blackbird (*Turdus merula* Linnaeus, 1758) and measured several song parameters. We then ran simple linear regression models and pairwise correlations for each species to analyze the effect of urbanity as well as of weather parameters on song parameters.

So far, few studies have examined song parameter variability in the blue tit (Doutrelant, Blondel, Perret, & Lambrechts, 2000; Doutrelant & Lambrechts, 2001; Doutrelant, Lambrechts, Giorgi, & Leitao, 1999), and the influence of the weather has only been investigated for singing behavior, not for song trait variability (Elkins, 2004). For the song analysis, we differentiate between two groups of song parameters. There are frequency parameters such as the frequency range (the bandwidth), frequency minima and maxima. Additionally, there are compositional or structural parameters such as durations or the amount of elements within a verse (Tietze et al., 2015) (Table 1).

**Table 1:** Song parameter definitions and how data were obtained (A = Automatically, M = Manually, C = Calculated)

Category	Parameter	Unit	Description	Source
	PCsong1		Principal component 1 for all frequency and structure parameters	C
	PCsong2		Principal component 2 for all frequency and structure parameters	C
Frequency	max.freq.	kHz	Maximum frequency within verse	M
	min.freq.	kHz	Minimum frequency within verse	M
	mean.freq.	kHz	Mean frequency within verse	C
	bandwidth	kHz	Bandwidth of the verse	A
	freq.trend.h	kHz	Upper frequency trend, difference between first and last high point within verse	C
	freq.trend.l	kHz	Lower frequency trend, difference between first and last low point within verse	C
	freq.trend.hAbs	kHz	Absolute upper frequency trend, freq.trend.h without sign	C
	freq.trend.lAbs	kHz	Absolute lower frequency trend, freq.trend.l without sign	C
	max.freq.el	kHz	Delta frequency of the element with the maximum bandwidth within verse	M
	min.freq.el	kHz	Delta frequency of the element with the minimum bandwidth within verse	M
	PCfreq1		Principal component 1 for all frequency parameters	C
	PCfreq2		Principal component 2 for all frequency parameters	C
Structure	number.el		Number of elements within verse	M
	number.el.typ		Number of element types within verse	M
	max.dur.el	s	Duration of longest element	M
	min.dur.el	s	Duration of shortest element	M
	duration	s	Duration of the verse	A
	speed	s^−1^	Speed of the verse as the number of elements divided by the duration of the verse	C
	PCstruct1		Principal component 1 for all structure parameters	C
	PCstruct2		Principal component 2 for all structure parameters	C

Specifically, we examined whether in the urban area, the minimal frequency within a verse is positively correlated with the volume of the ambient noise, as previously found in other cities, and if this effect can not only be shown for the great tit (Salaberria & Gil, 2010; Slabbekoorn & Peet, 2003) and the blackbird (Hu & Cardoso, 2010), but also for the blue tit. The song of the blackbird and the great tit covers a low‐to‐medium frequency range (1.5–6.2 kHz, Table S1), and the song of the blue tit covers a medium‐to‐high frequency range (4–8.4 kHz, Table S1). Therefore, our hypotheses are the following:

1. We expect a higher minimal frequency in the songs of great tits and blackbirds at higher levels of ambient noise.
2. We do not expect an upwards shift in the minimum frequency at higher levels of ambient noise for the blue tit as its high‐pitched song might not be affected by the masking effect of the low‐pitched ambient noise.

Noise and the degree of urbanity might not be the only drivers for song parameter variability. Generalist species, such as the three species we examined, find good habitats in cities with supplementary food resources, secure nesting sites, and less predation (Bókony et al., 2012; Evans, Chamberlain, Hatchwell, Gregory, & Gaston, 2011; Francis, Ortega, & Cruz, 2009; Lancaster & Rees, 1979; Morelli, Beim, Jerzak, Jones, & Tryjanowski, 2014). Therefore, weather could instead play a major role in song production of birds, and we developed a hypothesis how weather parameters could influence birdsong. With rising air temperatures, birds need less energy to maintain their body temperature (Marshall, 1961) and additionally, with rising temperatures, more food becomes available. As long as the supplementary energy and necessary diet for the offspring is not available, singing a more elaborate song might be an unnecessary cost. With improved conditions, birds may be able to increase their frequency range, the bandwidth of the song.

- We hypothesize that the blackbird should show a positive correlation between the bandwidth of a verse and the minimum air temperature.

This hypothesis can also be applied to the song of the blue and the great tit, but for reasons of clarity, we will focus on the results and discussion for the blackbird.

## 2 MATERIAL AND METHODS

### 2.1 Data acquisition and measurements

The investigated species all live throughout the city of Frankfurt as well as in the forests next to the city. Their breeding periods overlap, so they sing approximately at the same time of year (Bauer, 2012). We recorded only male individuals as ensured by visual identification during the recording.

**Figure 1:**
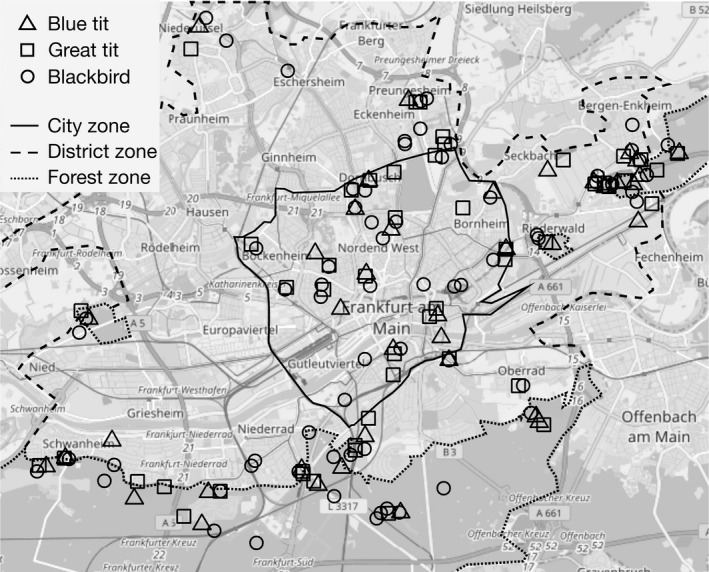
Map of Frankfurt am Main with the recording sites of the three species (symbols) and the division into three zones (lines). See legend above

Frankfurt, situated in the Lower Main Plain, is one of the biggest cities in Germany with a population of over 700,000 (City of Frankfurt am Main 2015‐10‐31). It has many highways (see Figure 1) and a highly frequented airport nearby (Gil, Honarmand, Pascual, Perez‐Mena, & Macías García, 2015). The average temperature in January is around 2 °C, the average temperature in July is above 19 °C, and the average annual temperature is above 10 °C. In the area of the Lower Main Plain, precipitation is low with 600–800 mm per year (Gedeon, Grüneberg, & Mitschke, 2014; Hessian National Office for Environment and Geology 2013).

To generate an urbanity gradient from urban to rural, we divided Frankfurt into three zones: city, district (the districts surrounding the city, but not the suburbs), and forest (the forests next to the city) (Figure 1). We never recorded twice at any recording site. Differences in the degree of urbanity between the zones are represented in the volume of the noise and in the principal components of the urbanity gradient containing information on the amount of impervious surface, further on referred to as the sealing off, and the building density and height (Table S2).

The recording period was from 2nd March to 13th June of 2015, that is, from spring to beginning of summer, which covers the breeding season of the three species. Recordings were made with a Telinga^®^ Pro6 microphone with a 2‐mm‐thick stationary dish (22” diameter) connected to a Marantz^®^ PMD660 Portable Solid State Recorder. As recording format, PCM‐44.1 K was chosen with a mono recording channel and a bit rate of 705.6 K. A total of 235 recordings were made, but only recordings with a sufficient number of verses were kept and measured: 39 of the blue tit, 50 of the great tit, and 71 of the blackbird. For each week, at least one recording was measured per species and per zone. The differing number of measured recordings is due to the fact that the tits, especially the blue tit, ceased singing earlier during the recording period.

All sonagraphic measurements were performed with the software Raven Pro 1.5 (Bioacoustics Research Program 2014). For all recordings, we used a Hamming window with a window size of 256 samples and 50% time grid overlap. The window frame was set to 0–11 kHz, and to 0–3 s, so that the spectrogram detail was always the same. The measurement frames were set manually. The duration and the bandwidth were noted automatically. All other song parameters were measured manually or calculated (Table 1). For the tits, we measured five verses and, for the blackbird, we measured ten verses per male due to its versatile song.

Additional data were collected at each recording site: coordinates for each site using a Garmin^®^ GPSmap 62 and the zone and volume of the ambient noise with a Voltacraft^®^ SL‐50 sound level meter. Urban variables that were collected included the degree, from 0 to 4, of the sealing off in a radius of 10 m around the recording site, of the building density in a radius of 50 m, and the number of floors of the highest building in the building density area. Furthermore, we noted the number of other birds singing during the recording as the number of competitors.

The map in Figure 1 was created with the “OpenStreetMap” package (Fellows & Stotz, 2013) in R version 3.2.1 (R Development Core Team 2015). The lines of the zones were added afterwards.

To examine the influence of the weather on the songs, we downloaded data of different weather parameters (Table 2) from the database of the Germany’s National Meteorological Service (Deutscher Wetterdienst, www.dwd.de) for the recording period. We chose data from weather station 1420, which is situated at Frankfurt airport. The values were modified by summing up the millimeters of precipitation and the hours of sunshine during a given day. All other hourly values were averaged per day. We also calculated the minimum and maximum temperature per day for soil and air.

**Table 2:** Explanatory parameter definitions

Category	Parameter	Unit	Description
Urbanity	Seal.Off	0–4	Sealing off in quarters of a circle of 10 m around singer
	Build.Dens.	0–4	Building density in quarters of a circle of 50 m around recording site
	Build.Height		Number of floors (from ground level) of the highest building in building density area
	PCug1		Principal component 1 for sealing off, building density and height
	PCug2		Principal component 2 for sealing off, building density and height
	Zone	C/D/F	Zone where recording was made
	Volume	dB	Volume of ambient noise
Weather	Wind	m/s	Mean wind speed per day
Data base:	Humidity	%	Mean atmospheric humidity per day
Climate	Precip.	mm	Sum of precipitation per day
Data Center,	Air.Press.	hPa	Mean air pressure per day
Values modified	Cloud.	0–8	Mean cloudiness per day
	Air.Temp.	°C	Mean air temperature per day
	Min.Air.Temp.	°C	Minimum air temperature per day
	Max.Air.Temp.	°C	Maximum air temperature per day
	Soil.Temp.	°C	Mean soil temperature per day, measured 5 cm below surface
	Min.Soil.Temp.	°C	Minimum soil temperature per day, measured 5 cm below surface
	Max.Soil.Temp.	°C	Maximum soil temperature per day, measured 5 cm below surface
	Sunshine	h	Sum of sunshine hours per day
	PCwe1		Principal component 1 for all weather parameters
	PCwe2		Principal component 2 for all weather parameters
Other	Day		Number of recording day (Julian Date)
	Daytime	AM/PM	Daytime when recording was made, before or after noon
	Compet.		Number of other birds singing while recording

### 2.2 Statistics

Statistics were performed in R 3.1.2 (R Development Core Team 2014). The five or ten verses, which were measured, were aggregated for each male bird into the mean of every song variable. Several principal component analyses were carried out separately for each species to reduce the parameters by getting principal components that covered most of the variance of the parameters. Therefore, we performed principal component analyses for all song parameters, for all frequency, structure, urbanity, and weather parameters (Tables 1 and 2). The principal component analysis for the urbanity parameters was based on the “degree of urbanity” introduced by Ziege et al. (2015) and was adjusted with relevant urbanity parameters for birds, like the building density and height.

We always extracted the first and the second principal component. In most cases, when combined, the first and second principal components explained over 50% of the variance of the corresponding variables (Table S3).

We performed a simple linear regression model for each song parameter for each species. We included all explanatory variables (Table 2) in the original model except for the principal components because of autocorrelation. We then chose a minimal model for each song parameter by performing a stepwise reduction of the explanatory variables, which had been put into the source model, using the AIC (Akaike Information Criterion). Only the models with the lowest AIC were kept for interpretation.

We also performed 528 correlation analyses for each species: we tested the correlation between each of the 22 song parameters and each of the 24 explanatory variables. Because of these multiple comparisons, we used Bonferroni's correction for all *p* values, further on referred to as p* (Armstrong, 2014; Streiner & Norman, 2011). All of the tested explanatory variables were continuous except for the daytime, which was a two‐level categorical variable. Therefore, we calculated Pearson's rank correlations and for the daytime Wilcoxon tests.

## 3 RESULTS

For the means and standard deviation of the song parameters for all three species and for the output of all pairwise correlations, see Tables S1 and S4–S7.

Concerning the first hypothesis, we expected that the song of the great tit and of the blackbird would encounter an increase in minimal frequency with a high volume of the ambient noise. Instead, for both species, the volume of the ambient noise had been discarded for the respective minimal models. Likewise, pairwise correlations showed for both species non‐significant relationships between the volume of the ambient noise and the minimum frequency within a verse (Pearson's rank correlation: for the great tit *r* = −.11, p* = 10.61; for the blackbird *r* = .01, p* = 22.46).

**Figure 2:**
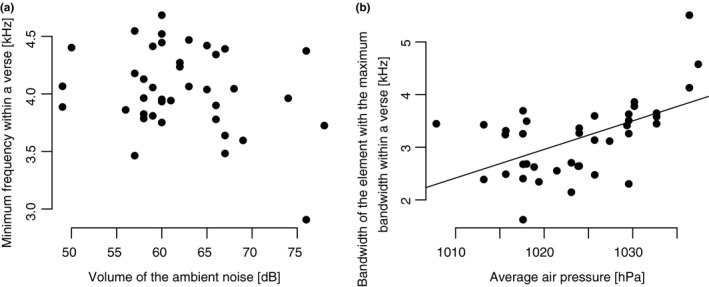
Correlation plots for the blue tit. (a) Non‐significant relationship between the volume of the ambient noise and the minimum frequency within a verse (*r* = −.27, p* = 2.4, *n* = 39). (b) Increasing frequency range of the element with the maximum bandwidth within a verse with increasing air pressure (p* = .008, *r* = .54, *n* = 39)

Concerning the second hypothesis, we expected that the volume of the ambient noise would not affect the minimum frequency of the blue tit's song. The volume of the ambient noise remained in the minimal model of the minimum frequency with a significant *p*‐value (*b* = −21.51, *t* = −3.55, *p* = .001), but the minimum air temperature (*b* = 182.01, *t* = 4.8, *p* < .001) and the atmospheric humidity (*b* = 32.72, *t* = 4.01, *p* < .001) had even higher *p*‐values. Pairwise correlation between the volume of the ambient noise and the minimum frequency of the verse revealed a non‐significant relationship (Pearson's rank correlation: *r* = −.27, p* = 2.4) (Figure 2a). Pairwise correlation with the minimum air temperature and the atmospheric humidity, respectively, showed non‐significant relationships.

Concerning the third hypothesis, we expected a broader bandwidth with increasing air temperature. The average air temperature did not remain in the minimal model of the bandwidth within a verse of the blackbird's song. Likewise, pairwise correlation between the average air temperature and the bandwidth showed a non‐significant relationship (Pearson's rank correlation: *r* = .32, p* = .15).

General results and representative examples of important results per species are shown in the following subchapters.

### 3.1 The blue tit

The minimal linear models for each song parameter of the blue tit contained urbanity and weather parameters with significant *p*‐values. In most cases, the weather parameters had higher significance levels than the urbanity parameters. The variables with the lowest *p*‐values in the different models mostly were the soil temperatures, the air temperatures, and the air pressure (Table 3).

**Table 3:** Output of the minimal linear regression models per species and per song parameter. As explanatory variables, only the most significant parameters from the model are represented. The adjusted *r*², the F‐statistic *F*, the degrees of freedom *df* for the model, and the residuals and the *p*‐value of the model are indicated in the four last columns

Species	Song parameters	Explanatory parameters	*r*²	*F*	*df*	*p*
Blue tit	PCsong1	Max.Soil.Temp., Air.Press., Max.Air.Temp.	.57	3.225	11, 27	.006
	PCsong2	Day, Min.Soil.Temp., Min.Air.Temp.	.71	4.786	13, 25	4 × 10^−4^
	max.freq.	Humidity, Max.Soil.Temp.	.25	2.843	7, 31	.021
	min.freq.	Min.Air.Temp., Humidity	.65	7.493	11, 27	1 × 10^−5^
	mean.freq.	Sunshine, Min.Air.Temp.	.40	4.192	8, 30	.002
	bandwidth	Day, Humidity, Soil.Temp.	.62	5.163	9, 29	3 × 10^−4^
	freq.trend.h	Max.Soil.Temp.	.50	6.689	5, 33	2 × 10^−4^
	freq.trend.l	Soil.Temp.	.49	6.255	5, 33	3 × 10^−4^
	freq.trend.hAbs	Max.Soil.Temp., Air.Press.	.50	6.682	5, 33	2 × 10^−4^
	freq.trend.lAbs	Soil.Temp., Air.Press.	.45	5.318	5, 33	.001
	max.freq.el	Air.Press., Humidity, Max.Air.Temp.	.65	3.987	12, 26	.002
	min.freq.el	Air.Press., Build.Dens.	.49	1.470	15, 23	.197
	PCfreq1	Max.Soil.Temp., Air.Press., Sunshine	.51	6.868	5, 33	2 × 10^−4^
	PCfreq2	Day, Min.Air.Temp., Soil.Temp.	.60	6.682	7, 31	7 × 10^−5^
	number.el	Min.Air.Temp., Air.Temp., Soil.Temp.	.50	3.286	9, 29	.007
	number.el.typ	Day, Min.Soil.Temp.	.61	3.389	12, 26	.004
	max.dur.el	Soil.Temp., DaytimePM	.52	3.529	9, 29	.005
	min.dur.el	Air.Temp., Min.Soil.Temp.	.54	5.160	7, 31	6 × 10^−4^
	duration	Air.Temp., Min.Air.Temp., Soil.Temp.	.52	3.497	9, 29	.005
	speed	Min.Soil.Temp.	.62	2.789	14, 24	.013
	PCstruct1	Day, Min.Soil.Temp., Min.Air.Temp.	.66	3.293	14, 24	.005
	PCstruct2	DaytimePM	.58	2.959	12, 26	.010
Great tit	PCsong1	Min.Air.Temp., Humidity	.45	4.208	8, 41	.001
	PCsong2	Sunshine, Max.Soil.Temp.	.45	4.919	7, 42	4 × 10^−4^
	max.freq.	Min.Air.Temp., Soil.Temp.	.41	2.745	10, 39	.012
	min.freq.	Sunshine, Humidity, Soil.Temp.	.33	3.519	6, 43	.006
	mean.freq.	Min.Air.Temp., Wind	.24	2.219	6, 43	.059
	bandwidth	Min.Air.Temp., Humidity, Min.Soil.Temp.	.51	3.985	10, 39	8 × 10^−4^
	freq.trend.h	Max.Soil.Temp., Day, Sunshine	.20	2.822	4, 45	.036
	freq.trend.l	Max.Soil.Temp., Day	.16	2.965	3, 46	.042
	freq.trend.hAbs	Sunshine, Min.Air.Temp., Air.Temp.	.35	3.242	7, 42	.008
	freq.trend.lAbs	Air.Temp., Min.Air.Temp., Sunshine	.43	2.959	10, 39	.007
	max.freq.el	Air.Press., Soil.Temp., Max.Soil.Temp.	.50	4.369	9, 40	5 × 10^−4^
	min.freq.el	Max.Air.Temp., Soil.Temp.	.46	3.377	10, 39	.003
	PCfreq1	Min.Air.Temp., Min.Soil.Temp.	.39	3.335	8, 41	.005
	PCfreq2	Sunshine	.38	3.749	7, 42	.003
	number.el	Build.Height, Min.Soil.Temp.	.38	3.627	7, 42	.004
	number.el.typ	Min.Soil.Temp., Max.Soil.Temp.	.55	4.261	11, 38	4 × 10^−4^
	max.dur.el	Day, Zone, Soil.Temp.	.68	5.800	13, 36	1 × 10^−5^
	min.dur.el	Day, DaytimePM, Humidity	.62	4.448	13, 36	2 × 10^−4^
	duration	Min.Soil.Temp.	.20	5.771	2, 47	.006
	speed	Min.Air.Temp., Humidity	.60	5.931	10, 39	2 × 10^−5^
	PCstruct1	Humidity	.53	4.390	10, 39	4 × 10^−4^
	PCstruct2	Max.Soil.Temp.	.58	4.839	11, 38	1 × 10^−4^
Blackbird	PCsong1	Day	.30	7.025	4, 66	9 × 10^−5^
	PCsong2	Wind, Day	.36	3.039	11, 59	.003
	max.freq.	Soil.Temp.	.28	5.086	5, 65	5 × 10^−4^
	min.freq.	DaytimePM	.31	4.064	7, 63	.001
	mean.freq.	Soil.Temp.	.27	4.759	5, 65	9 × 10^−4^
	bandwidth	Soil.Temp.	.31	4.903	6, 64	4 × 10^−4^
	freq.trend.h	Max.Soil.Temp.	.30	4.495	6, 64	7 × 10^−4^
	freq.trend.l	Min.Soil.Temp.	.19	7.985	2, 68	8 × 10^−4^
	freq.trend.hAbs	Soil.Temp.	.22	9.630	2, 68	2 × 10^−4^
	freq.trend.lAbs	Min.Soil.Temp.	.21	8.900	2, 68	4 × 10^−4^
	max.freq.el	DaytimePM	.14	2.712	4, 66	.037
	min.freq.el	Sunshine, Volume	.31	2.362	11, 59	.017
	PCfreq1	Soil.Temp.	.29	4.433	6, 64	8 × 10^−4^
	PCfreq2	Min.Soil.Temp.	.24	4.069	5, 65	.003
	number.el	Day, DaytimePM, Wind	.43	5.907	8, 62	1 × 10^−5^
	number.el.typ	Day, Wind	.44	6.011	8, 62	1 × 10^−5^
	max.dur.el	DaytimePM, Cloud., Min.Soil.Temp.	.25	2.005	10, 60	.048
	min.dur.el	Day, Compet.	.31	2.640	10, 60	.010
	duration	DaytimePM, Soil.Temp.	.40	4.535	9, 61	1 × 10^−4^
	speed	Min.Soil.Temp.	.15	3.851	3, 67	.013
	PCstruct1	Day, Wind	.36	3.306	10, 60	.002
	PCstruct2	DaytimePM, Volume	.31	5.739	5, 65	2 × 10^−4^

The explanatory variables which stayed most often in the minimal models of the song parameters with significant *p*‐values were the maximum and average soil temperatures and the air pressure. Variables which also remained quite often in the minimal models were the air and soil temperatures as well as the following urbanity parameters: the degree of sealing off and building density and the volume of the ambient noise.

The maximum soil temperature remained most often in the minimal models of frequency song parameters followed by air pressure, average soil temperature, and atmospheric humidity. Mostly, temperature variables and the degree of sealing off and building density remained in the minimal models for the structure song parameters.

Pairwise correlation between the most important variables of a model and the corresponding song parameter showed, for example, a highly significant correlation between the air pressure and the element with the maximum bandwidth within a verse (Pearson's rank correlation: *r* = .54, p* = .008) (Figure 2b). The frequency range of the element with the maximum bandwidth within a verse increased with the average air pressure.

### 3.2 The great tit

The minimal linear models for each song parameter of the great tit contained urbanity and weather parameters with significant *p*‐values. The variables with the lowest *p*‐values in the different models mostly were weather parameters such as air and soil temperatures but also the amount of sunlight per day and the atmospheric humidity (Table 3).

The explanatory variables which stayed most often in the minimal models of the song parameters with significant *p*‐values were the atmospheric humidity, the maximum soil temperature, and the minimum air temperature. Variables which also remained quite often in the minimal models were the soil and air temperatures as well as the building density and the number of hours of sunshine per day.

The maximum soil and minimum air temperature remained most often in the minimal models of frequency song parameters. Mostly, the atmospheric humidity remained in the minimal models for the structure song parameters.

**Figure 3:**
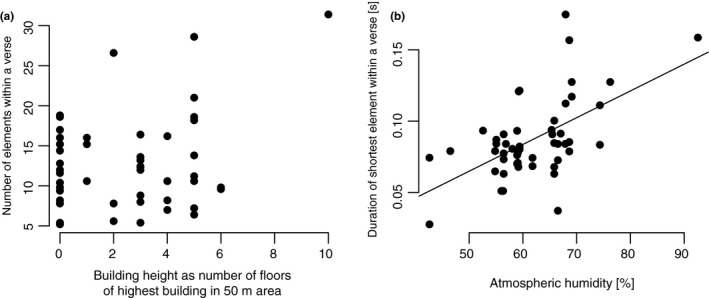
Correlation plots for the great tit. (a) Non‐significant correlation of the building height with the number of elements within a verse (*r* = .23, p* = 2.75, *n* = 50). (b) Rising duration of the shortest element within a verse with increasing average atmospheric humidity per day (*r* = .57, p* < .001, *n* = 50)

In only one model among all species, an urbanity parameter was the most important variable: The building height explained most of the variation of the number of elements in a verse of the great tit's song (*b* = 2.16, *t* = 3.35, *p* = .002). This relationship was not significant when performing a pairwise correlation (Pearson's rank correlation: *r* = .23, p* = 2.75) (Figure 3a).

Pairwise correlation between the other most important variables of a model and the corresponding song parameter showed, for example, a highly significant correlation between the atmospheric humidity and the duration of the shortest element within a verse (Pearson's rank correlation: *r* = .57, p* < .001) (Figure 3b). The duration of the shortest element within a verse increased with the mean atmospheric humidity.

### 3.3 The blackbird

The minimal linear models for each song parameter of the blackbird mostly contained weather parameters and only few urbanity parameters with significant *p*‐values. The soil temperature parameters were the variables with the lowest *p*‐values in most of the minimal models (Table 3).

The explanatory variables which stayed most often in the minimal models of the song parameters with significant *p*‐values were the average soil temperature and the daytime. Variables which also remained quite often in the minimal models were the wind speed, the minimum soil temperature, and the number of hours of sunlight per day. Urbanity parameters rarely remained in the minimal models with significant *p*‐values.

The average soil temperature and the daytime remained almost equally often in the minimal models of frequency and structure song parameters. The wind speed mainly remained in the minimal models for the structure song parameters.

**Figure 4:**
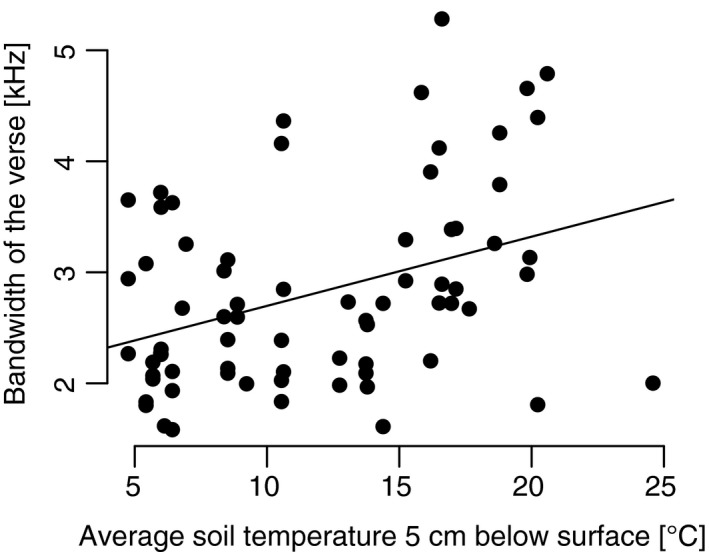
Correlation plot for the blackbird. Increasing bandwidth of a verse with rising average soil temperature 5 cm below the ground surface (*r* = .37, p* = .03, *n* = 71)

Pairwise correlation between the most important variables of a model and the corresponding song parameter showed, for example, a significant positive correlation between the mean soil temperature and the bandwidth of the verse (Pearson's rank correlation: *r* = .37, p* = .03) (Figure 4). The bandwidth of the verse increased with the mean soil temperature.

## 4 DISCUSSION

The volume of the ambient noise had no effect on the minimum song frequency for the blackbird and the great tit, and for the blue tit, it only plays a minor role in the minimal model. The pairwise correlation is not significant. Thus, hypotheses 1 and 2 can be rejected, that is, the minimum frequency in the songs of great tits and blackbirds is not higher at higher levels of ambient noise; and there is a slight albeit not significant downwards shift in the minimum frequency for the blue tit under ambient noise.

This might be because birds in Frankfurt generally face high noise pollution due to traffic, construction, planes, and highways in all three zones with an average of 60 ± 1 dB in each zone (Table S2). Gil et al. (2015) found that birds living near airports sing earlier in the morning and hence avoid the time of the first high noise event, which might also apply for the whole area of Frankfurt. This effect could be increased by artificial illumination, which is also supposed to lead to an earlier morning chorus (Kempenaers, Borgström, Loës, Schlicht, & Valcu, 2010). As both of these conditions exist for the entire Frankfurt study area, the three study species might avoid an overlap with noise by singing earlier. Further studies are needed to examine whether the investigated species advance their dawn chorus in comparison to conspecifics living at the same latitude, but in more quiet habitats.

Over all, the investigated urbanity parameters have a minor influence on song trait variability. They sometimes remain in the minimal models, but they rarely have low *p*‐values, and the pairwise correlations between song and urbanity parameters often are not significant at all.

The fact that the other investigated urbanity parameters besides the ambient noise do not show a great effect on the song parameters suggests that the city might be a favorable habitat, at least for the investigated species (Lancaster & Rees, 1979), although providing different and supposedly harsher conditions than natural environments (Table S2) (Chamberlain et al., 2009). Maklakov, Immler, Gonzalez‐Voyer, Rönn, and Kolm (2011) suggest that species with relatively big brains adapt or cope better with the conditions of urban environments. Members of the Paridae have relatively big brains (Maklakov et al., 2011) and therefore might succeed better in urban areas, which would support our findings for the blue and great tits. The reason for the success of European blackbirds in colonizing urban areas remains unclear, but higher temperatures and a greater food supply might play a major role (Evans, Hatchwell, Parnell, & Gaston, 2010).

Previous studies have already shown that weather does have an impact on breeding, feeding, singing behavior, and on avian life cycles (Elkins, 2004; Poesel, Kunc, Foerster, Johnsen, & Kempenaers, 2006; Slagsvold, 1977). This consequently raises the question why weather should not also have an impact on the song itself. For our three investigated species, we found many highly significant weather variables remaining in the minimal models as well as several highly significant correlations with weather parameters. Hence, it seems that weather parameters are more important for song trait variability. These findings including hypothesis (3) are discussed in the following sections.

### 4.1 The blue tit

Along with the temperatures, the air pressure has a profound influence on the blue tit's song trait variability. There is not much known about how air pressure modulates sounds and thus birdsong, but it is known that with decreasing air pressure also the oxygen partial pressure decreases, that is, the lower the air pressure, the less oxygen in the air. Considering the lower oxygen partial pressure in the air, one might hypothesize that birds experiencing low air pressure would have a simpler song to ensure oxygen supply. The blue tit has a narrower bandwidth of the element with the maximum bandwidth within a verse, when the air pressure is low. Hence, our findings would support this conclusion.

There have been studies on bird song along elevational gradients, but they did not investigate the effect of the air pressure, and they did not compare within‐species variability but compared congeneric species, or species within a subfamily (Caro, Caycedo‐Rosales, Bowie, Slabbekoorn, & Cadena, 2013; Jankowski, Robinson, & Levey, 2010; Snell‐Rood & Badyaev, 2008). At this stage, there is no simple explanation why the three species in our study react differently to changes in air pressure. Therefore, there is a need for further investigation to better understand how air pressure modulates sound and which effect it has on different song traits and their transmission and if species with similar song characteristics show similar changes.

In the case of the blue tit, hypothesis 3, that the bandwidth of the song widens with increasing air temperature, can be rejected. The bandwidth of their song is not influenced by air temperature, but by other weather parameters such as atmospheric humidity and average soil temperature.

### 4.2 The great tit

The atmospheric humidity is one of the most important variables in the minimal models for the great tit along with soil and air temperature variables and the amount of sunlight per day. The atmospheric humidity plays a more important role in the models of the structure parameters as in the minimal model for the duration of the shortest element within a verse. Briefly, with increasing atmospheric humidity, the elements become longer.

Harris (1966) and Gomez‐Augustina, Dance, and Shield (2014) describe the effects of air temperature and atmospheric humidity on sound attenuation and reverberation times. In general, high frequencies are absorbed the most and even more so when atmospheric humidity is low. Reverberation time is low when frequencies are high and when atmospheric humidity is low. The great tit, which has a mean frequency of about 4.6 kHz (Table S1), is situated in a medium frequency range and therefore less affected by sound attenuation. But as reverberation time increases with lower frequencies along the atmospheric humidity, it might be an explanation for the importance of the atmospheric humidity in the minimal models of the great tit as well as the highly significant correlation of the duration of the shortest elements within a verse with the humidity.

The great tit has longer elements within a verse when humidity is high, and hence, the elements have high reverberation times (for graphics see Harris (1966) and Gomez‐Augustina et al. (2014)). The high reverberation time might favor the sound transmission and might facilitate song perception by females (Slabbekoorn, Ellers, & Smith, 2002). Some of the great tit's song types might be defined as narrow frequency bandwidth notes as described by Slabbekoorn et al. (2002) and might show these benefits from reverberation.

In the case of the great tit, hypothesis 3—increasing air temperatures supposedly leading to a wider bandwidth of the song—can be rejected. The minimum air temperature stays in the minimal model for the bandwidth of the song; nevertheless, the direct correlation is not significant. The bandwidth of the song might not be an appropriate song parameter for identifying the influence of weather parameters on the great tit's song. This might be due to the fact that the latter is grouped into motifs that are repeated, but mostly stay within a certain frequency range in contrast to, for example, the versatile song of the blackbird.

### 4.3 The blackbird

Soil temperatures play a highly important role in the minimal models of the blackbird. Coming back to hypothesis 3 suggesting a positive relationship between the minimum air temperature and the bandwidth of the verse, we found that the minimum air temperature was discarded in the stepwise selection of the minimal model. Instead, the minimum soil temperature turned out to be the most important variable in the minimal model for the bandwidth within a verse. Briefly, with increasing minimum soil temperature, the bandwidth of the verse widens.

It seems that with warmer temperatures 5 cm below the ground surface, blackbirds have more energy for a more elaborate song. With warmer temperatures, they need less energy to sustain their body temperature and they might get additional energy from food sources below the ground, especially as the European blackbird mainly feeds on earthworms and caterpillars (Tomialojc, 1994). Regarding earthworms, they can pull them out of the ground more easily as soon as the soil warms up and becomes softer.

Birds normally singing in a low‐frequency range might need more energy for singing in a wider frequency range and males that succeed in wider bandwidths might indicate a higher physical fitness and/or better nutrition. Both would be aspects a female might select for during courtship.

## 5 CONCLUSION

Summarizing, we found that temperature variables play an important role for all of the three investigated species, but also other weather parameters such as air pressure, atmospheric humidity, but also the amount of sunshine and wind seem to influence song trait variability. Urbanity parameters sometimes remain in the minimal models with significant *p*‐values, but they seem to be less important than weather parameters. We found a tendency that the smaller the study species (body mass means: blue tit 11.7 g, great tit 19 g, blackbird 86 g; Glutz von Blotzheim, Bauer, Haffer, van den Elzen, & Grüll, 1993), the higher the coefficients of determination of the models (*r*² means: blue tit 0.54, great tit 0.43, blackbird 0.29) (Pearson's product‐moment correlation: *r* = −0.93, *p* = .23, *n* = 3). As the models are mostly fitted with weather parameters, this indicates that smaller birds might have a stronger dependency on weather parameters.

Regarding the influence of meteorological variables on song traits, it seems that different species show different song adaptations, as unlike our findings, Brumm (2004) found no effect of environmental influences on song variables of the nightingale, *Luscinia megarhynchos* (C. L. Brehm, 1831), when considering air temperature and atmospheric humidity.

To conclude, we could show that song parameter variability for the three investigated species is driven more by weather than by urbanity in the city of Frankfurt. Consequently, the findings raise further questions. Perhaps the three species are not only affected by climate change due to a change in vegetation and in temperatures (that have an influence on the food supply and the breeding biology of birds; Visser, Holleman, & Gienapp, 2006), but also by a direct effect on mate attraction and on the establishment and the defense of a territory. We therefore suggest that

1. weather parameters should be considered in future studies and it should be examined in more depth how they influence sound transmission and perception;
2. additional weather parameters should be tested, for example, the temperature or precipitation parameters from the previous day could influence the song whereas in this study, only daily means or sums of weather variables were considered;
3. this type of study should be replicated for other cities of comparable size in order to investigate, if the lack of correlations with the volume of the ambient noise is specific to Frankfurt because of high noise pollution throughout the city or to big cities in general;
4. earlier studies should be repeated to examine, if there have been changes, or further adaptations, in the bird populations investigated at that time;
5. and as already suggested by Nemeth and Brumm (2009), the extent to which hormones play a role in singing behavior (van Duyse, Pinxten, & Eens, 2003) and on song parameters should be examined further as well as how hormone production and balance may be different in urban compared to rural environments (Fokidis, Orchinik, & Deviche, 2009; Partecke, Schwabl, & Gwinner, 2006). There is also the possibility, as weather parameters seem to have an effect on hormone levels (Wingfield, Moore, & Farner, 1983), that weather parameters might in fact indirectly affect song parameters.

## CONFLICT OF INTEREST

The authors declare no conflict of interest.

## AUTHOR CONTRIBUTIONS

D.T.T. designed the study; M.M.J. and J.E.S. collected the data; D.T.T., M.M.J., and J.E.S. analyzed the data; S.K. provided financial support; J.E.S. wrote the initial manuscript draft; all authors wrote on the manuscript.
