# Supplementary material for: How weather instead of urbanity measures affects song trait variability in three European passerine bird species
Source: Ecol Evol. 2017 May 28;7(13):4868–80. doi: 10.1002/ece3.3032 (PMC5496528; doi:10.1002/ece3.3032)
Supplement: Supplementary file 1 [file ECE3-7-4868-s001.docx]

### **Supplementary Table 1**

Measurements of song parameters for three passerine bird species (mean and standard deviation; blue tit: n = 39, great tit: n = 50, blackbird: n = 71).

|  |  |  |  |
| --- | --- | --- | --- |
| **Song parameter** | **Blue tit** | **Great tit** | **Blackbird** |
| max.freq [kHz] | 8.4 ± 0.37 | 6.2 ± 0.97 | 4.3 ± 0.82 |
| min.freq [kHz] | 4.0 ± 0.36 | 3.1 ± 0.53 | 1.5 ± 0.12 |
| mean.freq [kHz] | 6.2 ± 0.24 | 4.6 ± 0.57 | 2.9 ± 0.39 |
| bandwidth [kHz] | 4.4 ± 0.55 | 3.0 ± 1.07 | 2.8 ± 0.87 |
| freq.trend.h [kHz] | -2.1 ± 0.95 | -0.01 ± 0.56 | 0.6 ± 0.35 |
| freq.trend.l [kHz] | -1.9 ± 1.11 | -0.01 ± 0.58 | 0.4 ± 0.30 |
| freq.trend.hAbs [kHz] | 2.2 ± 0.89 | 0.5 ± 0.29 | 0.6 ± 0.32 |
| freq.trend.lAbs [kHz] | 2.0 ± 0.97 | 0.5 ± 0.31 | 0.5 ± 0.25 |
| max.freq.el [kHz] | 3.2 ± 0.72 | 1.6 ± 0.59 | 1.9 ± 0.57 |
| min.freq.el [kHz] | 1.0 ± 0.43 | 0.8 ± 0.19 | 0.7 ± 0.10 |
| number.el | 9.9 ± 2.96 | 12.8 ± 5.76 | 7.1 ± 1.42 |
| number.el.typ | 2.8 ± 0.41 | 1.8 ± 0.57 | 6.7 ± 1.23 |
| max.dur.el [s] | 0.2 ± 0.05 | 0.2 ± 0.07 | 0.3 ± 0.07 |
| min.dur.el [s] | 0.1 ± 0.02 | 0.1 ± 0.03 | 0.1 ± 0.03 |
| duration [s] | 1.3 ± 0.27 | 2.3 ± 0.79 | 1.8 ± 0.38 |
| speed [s^-1^] | 7.5 ± 1.52 | 5.6 ± 1.35 | 4.0 ± 0.48 |

### **Supplementary Table 2**

Values of the urbanity gradient principal component and the volume of the mean ambient noise in the three zones of Frankfurt.

|  |  |  |  |  |  |
| --- | --- | --- | --- | --- | --- |
| **Explanatory variable** | **PCug1** |  |  |  | **Volume [dB]** |
| **Species** | all | Blue tit | Great tit | Blackbird |  |
| City zone | 1.46 | 1.55 | 1.33 | 1.50 | 61.15 |
| District zone | 0.57 | 1.10 | 0.49 | 0.39 | 59.76 |
| Forest zone | -1.95 | -1.71 | -2.11 | -2.04 | 60.23 |

### **Supplementary Table 3**

Proportion of variance for each principal component PC (first row) explained by first (1) and second (2) principal component in percent.

|  |  |  |  |  |  |  |
| --- | --- | --- | --- | --- | --- | --- |
| **Species** | **Blue tit** |  | **Great tit** |  | **Blackbird** |  |
|  | 1 | 2 | 1 | 2 | 1 | 2 |
| Urbanity gradient PC | 87.40 | 10.90 | 87.30 | 10.50 | 85.90 | 11.80 |
| Weather PC | 53.30 | 19.70 | 53.50 | 17.80 | 47.80 | 22.10 |
| Song PC | 34.10 | 25.10 | 27.90 | 18.80 | 45.80 | 19.20 |
| Frequency parameters PC | 48.30 | 25.50 | 32.50 | 24.90 | 67.20 | 13.70 |
| Structure parameters PC | 46.50 | 20.90 | 41.90 | 31.40 | 52.10 | 36.40 |

**Supplementary Tables 4 - 7** Correlation coefficients for all pairwise correlations. Values are r values and W values for the daytime. In red are the significant correlations after Bonferroni correction (Pearson’s rank correlation p* < 0.05), the asterisks indicate the significance level (*: p* < 0.05, **: p* < 0.01, ***: p* < 0.001). Values in bold stand for parameters, which remained in the minimal model of the respective song parameters with a significant p value, the parameter highlighted in yellow contributed most to the minimal model of the respective song parameter.

Abbreviations: Song par. = Song parameter. Table 3 - 4: Seal.Off = Sealing off; Build.Dens. Height = Building density, height; Compet. = Competitors. Table 5 - 6: Min. = Minimum; Max. = Maximum; Temp. = Temperature; Air.Press. = Air pressure; Precip. = Precipitation; Sunshine = Sunshine hours; Cloud. = Cloudiness. For definitions see Table 1 and 2.

**Supplementary Table 4** Correlation between song structure parameters and urbanity and other explanatory variables.

|  | Species | PCug1 | PCug2 | Seal.Off | Build.Dens. | Build.Height | Volume | Zone | Compet. | Day | Daytime | PCwe1 | PCwe2 |
| --- | --- | --- | --- | --- | --- | --- | --- | --- | --- | --- | --- | --- | --- |
| PCsong1 | Cc | 0.07 | -0.06 | 0.05 | 0.06 | 0.09 | 0.01 | -0.02 | -0.28 | -0.51* | 173 | -0.37 | 0.41 |
|  | Pm | 0.04 | -0.28 | 0.13 | **0.09** | **-0.09** | -0.07 | 0.07 | 0.01 | 0.25 | 359 | 0.15 | -0.15 |
|  | Tm | 0.07 | -0.17 | 0.12 | 0.1 | -0.02 | -0.16 | -0.1 | -0.13 | **0.42**** | **451** | 0.38* | -0.001 |
| PCsong2 | Cc | -0.07 | 0.16 | -0.03 | -0.03 | -0.14 | **-0.22** | -0.03 | -0.04 | **0.27** | 205 | 0.23 | -0.06 |
|  | Pm | 0.12 | 0.16 | 0.05 | 0.11 | 0.18 | 0.14 | -0.07 | -0.1 | -0.26 | 340 | -0.37 | -0.17 |
|  | Tm | 0.03 | 0.12 | -0.02 | 0.02 | 0.08 | **-0.13** | -0.05 | **0.14** | **-0.18** | 600 | -0.1 | 0.12 |
| number.el | Cc | 0.07 | -0.05 | 0.07 | 0.05 | 0.09 | **0.19** | 0.05 | -0.11 | -0.22 | 203 | -0.17 | 0.002 |
|  | Pm | 0.07 | 0.36 | -0.06 | **0.04** | **0.23** | 0.01 | -0.08 | -0.07 | -0.26 | 346.5 | -0.17 | -0.08 |
|  | Tm | 0.08 | -0.21 | 0.13 | 0.12 | -0.04 | -0.14 | -0.06 | **-0.22** | **0.32** | **432.5** | 0.22 | -0.08 |
| number.el.typ | Cc | -0.09 | -0.14 | **-0.12** | **-0.12** | **-0.02** | 0.05 | **0.17** | 0.11 | **-0.03** | 199 | -0.05 | -0.24 |
|  | Pm | 0.05 | 0.17 | **-0.01** | 0.04 | 0.12 | -0.08 | **-0.19** | **0.12** | -0.01 | 250.5 | -0.01 | 0.25 |
|  | Tm | 0.07 | -0.23 | 0.13 | 0.11 | -0.05 | -0.11 | -0.04 | **-0.21** | **0.32** | **452** | 0.25 | -0.06 |
| max.dur.el | Cc | -0.02 | 0.02 | -0.05 | 0.02 | **-0.04** | 0.05 | 0.02 | -0.05 | **0.16** | **247.5** | 0.22 | -0.15 |
|  | Pm | 0.21 | 0.3 | 0.06 | 0.22 | 0.3 | 0.36 | **-0.07** | -0.27 | **-0.51**** | 252 | -0.53** | 0.001 |
|  | Tm | 0.05 | 0.07 | -0.01 | 0.08 | 0.08 | -0.15 | -0.03 | -0.05 | -0.08 | **437** | -0.04 | 0.01 |
| min.dur.el | Cc | -0.31 | -0.05 | **-0.34** | **-0.27** | -0.26 | -0.05 | 0.22 | -0.003 | **-0.11** | 190.5 | -0.07 | -0.01 |
|  | Pm | 0.14 | 0.05 | 0.11 | 0.15 | 0.15 | 0.21 | **-0.05** | -0.25 | **-0.33** | **360.5** | -0.38 | -0.09 |
|  | Tm | 0.09 | 0.04 | 0.05 | **0.1** | 0.09 | -0.19 | -0.07 | **0.18** | **-0.24** | 568.5 | -0.19 | 0.13 |
| speed | Cc | 0.05 | -0.11 | **0.05** | **0.01** | **0.11** | **0.03** | **0.03** | 0.16 | **0.03** | 176 | -0.02 | 0.04 |
|  | Pm | -0.06 | 0.2 | -0.11 | **-0.1** | **0.04** | **-0.16** | -0.05 | **-0.01** | 0.05 | 329 | 0.21 | 0.1 |
|  | Tm | 0.001 | -0.14 | 0.05 | 0.01 | -0.07 | 0.1 | 0.05 | 0.02 | 0.31 | 740 | 0.23 | -0.12 |
| duration | Cc | 0.1 | -0.003 | 0.08 | 0.12 | 0.09 | 0.22 | -0.03 | -0.35 | -0.45 | 199 | -0.33 | -0.01 |
|  | Pm | 0.1 | 0.25 | -0.004 | 0.08 | 0.2 | 0.13 | -0.03 | -0.06 | -0.38 | 387 | -0.37 | -0.09 |
|  | Tm | 0.05 | -0.11 | 0.07 | 0.08 | -0.01 | -0.2 | -0.06 | **-0.22** | **0.05** | **336*** | 0.01 | -0.002 |
| PCstruct1 | Cc | -0.13 | 0.07 | **-0.13** | **-0.09** | **-0.15** | **-0.13** | **0.01** | 0.01 | **0.11** | 181 | 0.11 | 0.03 |
|  | Pm | 0.05 | -0.19 | 0.1 | **0.08** | -0.05 | 0.17 | 0.06 | -0.11 | -0.05 | 288 | -0.16 | -0.07 |
|  | Tm | 0.03 | -0.19 | 0.09 | 0.05 | -0.07 | -0.03 | -0.01 | -0.2 | **0.33** | **512** | 0.24 | -0.1 |
| PCstruct2 | Cc | 0.005 | 0.02 | **-0.02** | **0.05** | -0.01 | 0.2 | 0.02 | **-0.34** | -0.34 | **222** | -0.21 | -0.04 |
|  | Pm | 0.2 | 0.35 | 0.05 | 0.19 | **0.32** | 0.27 | -0.09 | -0.22 | **-0.54**** | **357** | -0.55*** | -0.07 |
|  | Tm | -0.08 | 0.02 | -0.05 | -0.11 | -0.05 | **0.24** | 0.08 | 0.13 | 0.07 | **915*** | 0.06 | -0.05 |

**Supplementary Table 5** Correlation between song frequency parameters and urbanity and other explanatory variables.

| Song par. | Species | PCug1 | PCug2 | Seal.Off | Build.Dens. | Build.Height | Volume | Zone | Compet. | Day | Daytime | PCwe1 | PCwe2 |
| --- | --- | --- | --- | --- | --- | --- | --- | --- | --- | --- | --- | --- | --- |
| max.freq. | Cc | 0.02 | 0.03 | 0.03 | 0.02 | 0.005 | 0.25 | -0.005 | 0.17 | -0.02 | 198 | -0.09 | -0.27 |
|  | Pm | -0.09 | 0.27 | -0.17 | **-0.13** | 0.05 | 0.07 | **-0.04** | 0.04 | **-0.24** | 264 | -0.2 | 0.06 |
|  | Tm | 0.13 | -0.1 | 0.14 | 0.14 | 0.06 | -0.17 | -0.14 | -0.17 | 0.4* | **436** | 0.33 | -0.07 |
| min.freq. | Cc | 0.07 | 0.37 | **0.21** | 0.08 | -0.1 | **-0.27** | -0.06 | -0.02 | **0.36** | **285** | 0.22 | -0.34 |
|  | Pm | -0.1 | -0.04 | -0.09 | -0.07 | -0.12 | -0.11 | 0.1 | 0.12 | 0.17 | 287 | 0.05 | -0.13 |
|  | Tm | -0.02 | 0.23 | **-0.09** | -0.04 | **0.1** | 0.01 | 0.02 | -0.11 | -0.22 | **879** | -0.18 | 0.04 |
| mean.freq. | Cc | 0.07 | 0.3 | 0.18 | 0.08 | -0.07 | -0.01 | -0.05 | 0.11 | 0.25 | 273 | 0.1 | -0.46 |
|  | Pm | -0.13 | 0.21 | -0.19 | -0.14 | -0.02 | 0.004 | 0.01 | 0.09 | -0.13 | 277 | -0.15 | -0.01 |
|  | Tm | 0.13 | -0.07 | 0.14 | 0.14 | 0.08 | -0.17 | -0.14 | -0.2 | 0.39* | 490 | 0.32 | -0.07 |
| max.freq.el | Cc | 0.12 | -0.13 | 0.07 | **0.11** | 0.16 | 0.09 | -0.03 | -0.18 | -0.53* | 154 | -0.38 | 0.45 |
|  | Pm | 0.02 | 0.001 | **0.04** | **-0.02** | **0.04** | **0.1** | -0.09 | -0.02 | **-0.21** | 280 | -0.18 | 0.27 |
|  | Tm | 0.05 | -0.13 | 0.07 | 0.09 | -0.02 | -0.17 | -0.11 | -0.1 | 0.11 | **419** | 0.09 | -0.01 |
| min.freq.el | Cc | 0.08 | 0.03 | **0.07** | **0.11** | **0.06** | 0.06 | -0.14 | -0.16 | 0.002 | 244 | 0.02 | 0.17 |
|  | Pm | -0.14 | -0.08 | -0.08 | **-0.17** | -0.15 | 0.32 | 0.05 | -0.11 | -0.16 | 300.5 | -0.18 | 0.2 |
|  | Tm | 0.1 | 0.03 | 0.09 | 0.08 | 0.1 | **-0.24** | -0.08 | 0.09 | **-0.16** | 489 | -0.13 | 0.03 |
| freq.trend.h | Cc | 0.12 | -0.07 | 0.1 | 0.09 | 0.14 | 0.08 | -0.06 | -0.31 | -0.54** | 188 | -0.42 | 0.34 |
|  | Pm | 0.03 | 0.02 | 0.02 | 0.02 | 0.04 | 0.1 | 0.02 | -0.12 | **0.05** | 279 | -0.03 | -0.2 |
|  | Tm | 0.02 | -0.14 | 0.06 | 0.05 | -0.05 | -0.17 | -0.07 | -0.07 | 0.41* | 587 | 0.43** | 0.09 |
| freq.trend.hAbs | Cc | -0.12 | 0.03 | -0.11 | -0.1 | -0.12 | -0.06 | 0.05 | 0.31 | 0.53* | 193 | 0.42 | -0.34 |
|  | Pm | -0.22 | 0.03 | -0.22 | -0.2 | -0.19 | 0.04 | 0.21 | -0.02 | 0.1 | 215 | 0.18 | 0.03 |
|  | Tm | 0.03 | -0.14 | 0.06 | 0.06 | -0.04 | -0.09 | -0.07 | -0.03 | 0.39* | 602 | 0.42** | 0.06 |
| freq.trend.l | Cc | 0.05 | -0.05 | 0.05 | 0.03 | 0.07 | -0.03 | 0.002 | -0.17 | -0.52* | 177 | -0.38 | 0.36 |
|  | Pm | -0.03 | 0.01 | -0.03 | -0.05 | -0.02 | 0.08 | 0.05 | -0.08 | **0.07** | 292 | -0.01 | -0.16 |
|  | Tm | -0.02 | -0.15 | 0.03 | -0.004 | -0.09 | -0.09 | -0.02 | -0.02 | 0.36* | 620 | 0.38* | 0.15 |
| freq.trend.lAbs | Cc | -0.02 | -0.06 | -0.04 | -0.02 | 0.01 | 0.08 | -0.08 | 0.17 | 0.51* | 204 | 0.37 | -0.34 |
|  | Pm | -0.14 | 0.06 | -0.16 | -0.13 | -0.11 | **0.1** | 0.15 | -0.03 | -0.0003 | **237** | 0.1 | 0.1 |
|  | Tm | -0.004 | -0.09 | 0.03 | 0.003 | -0.05 | -0.06 | -0.04 | 0.03 | 0.37* | 638 | 0.38* | 0.15 |
| bandwidth | Cc | -0.03 | -0.22 | -0.11 | -0.04 | 0.07 | **0.35** | 0.04 | **0.13** | **-0.25** | 134 | -0.21 | 0.05 |
|  | Pm | -0.03 | 0.27 | -0.11 | **-0.08** | 0.1 | 0.11 | **-0.09** | -0.03 | -0.3 | 272 | -0.21 | 0.12 |
|  | Tm | 0.12 | -0.13 | 0.15 | 0.14 | 0.04 | -0.16 | **-0.14** | -0.15 | 0.41** | **408** | 0.34 | -0.07 |
| PCfreq1 | Cc | 0.07 | -0.08 | 0.04 | 0.05 | 0.1 | -0.01 | -0.01 | -0.26 | -0.51* | 171 | -0.36 | 0.44 |
|  | Pm | 0.13 | -0.2 | 0.17 | **0.16** | 0.01 | -0.14 | **-0.01** | 0.02 | 0.23 | 373 | 0.15 | -0.15 |
|  | Tm | 0.07 | -0.14 | 0.1 | 0.09 | -0.01 | -0.16 | **-0.1** | -0.08 | 0.38* | **479** | 0.36* | 0.03 |
| PCfreq2 | Cc | -0.01 | -0.25 | -0.1 | -0.02 | 0.11 | **0.3** | 0.03 | **0.06** | **-0.37** | 129 | -0.28 | 0.19 |
|  | Pm | 0.01 | 0.1 | -0.02 | -0.001 | 0.05 | -0.03 | -0.02 | 0.02 | 0.03 | 303 | -0.08 | -0.2 |
|  | Tm | 0.11 | 0.02 | 0.1 | 0.11 | 0.11 | **-0.12** | -0.09 | -0.03 | -0.23 | **463** | -0.29 | -0.16 |

**Supplementary Table 6** Correlation between song structure parameters and weather variables.

|  | Species | Air.Temp. | Min.Air.Temp. | Max.Air.Temp. | Soil.Temp. | Min.Soil.Temp. | Max.Soil.Temp. | Air.Press. | Humidity | Precip. | Sunshine | Cloud. | Wind |
| --- | --- | --- | --- | --- | --- | --- | --- | --- | --- | --- | --- | --- | --- |
| PCsong1 | Cc | -0.35 | -0.4 | **-0.28** | -0.46 | -0.5* | **-0.38** | **0.54*** | 0.22 | -0.13 | 0.03 | -0.14 | -0.13 |
|  | Pm | 0.15 | **0.23** | **0.12** | 0.18 | **0.19** | **0.16** | -0.1 | **0.06** | 0.04 | 0.03 | 0.21 | 0.005 |
|  | Tm | 0.36 | 0.31 | 0.34 | 0.41* | 0.39* | 0.37* | 0.03 | -0.13 | 0.13 | 0.03 | 0.04 | -0.18 |
| PCsong2 | Cc | 0.26 | **0.27** | **0.3** | 0.22 | **0.19** | 0.23 | **-0.01** | **0.01** | 0.09 | 0.17 | 0.12 | -0.1 |
|  | Pm | -0.33 | -0.2 | **-0.39** | **-0.35** | -0.31 | **-0.4** | -0.1 | 0.36 | 0.24 | **-0.12** | -0.17 | 0.35 |
|  | Tm | -0.05 | -0.04 | -0.06 | **-0.1** | **-0.1** | -0.09 | **0.14** | 0.05 | -0.12 | **0.07** | **-0.22** | **0.09** |
| number.el | Cc | **-0.18** | **-0.19** | -0.22 | **-0.18** | **-0.14** | -0.19 | -0.01 | -0.04 | **-0.05** | -0.12 | -0.08 | **0.14** |
|  | Pm | -0.14 | -0.16 | -0.13 | -0.23 | **-0.23** | -0.22 | -0.19 | **-0.06** | 0.02 | -0.05 | -0.09 | 0.22 |
|  | Tm | **0.19** | 0.17 | 0.18 | **0.24** | 0.23 | 0.21 | 0.01 | **-0.07** | 0.21 | -0.05 | 0.16 | **-0.23** |
| number.el.typ | Cc | **-0.06** | 0.01 | -0.12 | -0.01 | **0.05** | **-0.06** | **-0.3** | -0.06 | 0.14 | -0.13 | -0.03 | 0.22 |
|  | Pm | -0.02 | **-0.1** | **0.02** | 0.01 | **0.01** | **0.01** | 0.32 | **-0.09** | -0.06 | -0.05 | -0.2 | -0.19 |
|  | Tm | 0.21 | **0.19** | 0.22 | **0.25** | 0.24 | 0.24 | **-0.03** | **-0.09** | 0.19 | -0.01 | 0.16 | **-0.24** |
| max.dur.el | Cc | **0.25** | 0.27 | 0.22 | **0.25** | 0.29 | **0.19** | -0.17 | **0.08** | 0.04 | **0.05** | 0.11 | -0.03 |
|  | Pm | **-0.47*** | -0.4 | -0.46* | **-0.49**** | -0.46* | **-0.53**** | **0.09** | **0.6***** | 0.1 | -0.35 | **-0.1** | 0.04 |
|  | Tm | -0.01 | 0.01 | -0.02 | **-0.06** | **-0.06** | -0.06 | -0.02 | 0.06 | 0.06 | **0.06** | **-0.15** | 0.09 |
| min.dur.el | Cc | **0.001** | 0.02 | 0.04 | -0.11 | **-0.15** | **-0.09** | **0.09** | 0.08 | 0.23 | 0.11 | -0.08 | 0.04 |
|  | Pm | -0.31 | -0.2 | **-0.36** | **-0.33** | -0.27 | -0.4 | 0.09 | **0.57***** | 0.08 | -0.4 | **0.12** | -0.03 |
|  | Tm | -0.15 | -0.11 | -0.16 | **-0.19** | -0.2 | -0.18 | **0.26** | 0.1 | -0.11 | 0.01 | -0.18 | **-0.03** |
| speed | Cc | -0.08 | **-0.1** | -0.15 | **0.01** | **0.06** | **-0.004** | 0.05 | -0.16 | -0.18 | -0.11 | 0.004 | **0.07** |
|  | Pm | **0.19** | **0.03** | 0.25 | 0.15 | 0.09 | 0.2 | -0.12 | **-0.39** | -0.11 | **0.21** | -0.09 | -0.08 |
|  | Tm | 0.19 | 0.15 | 0.17 | 0.27 | **0.29** | 0.24 | -0.18 | -0.07 | 0.05 | -0.12 | 0.14 | -0.09 |
| duration | Cc | **-0.27** | **-0.27** | **-0.27** | **-0.39** | -0.39 | -0.38 | -0.03 | 0.17 | 0.14 | -0.08 | -0.15 | 0.17 |
|  | Pm | -0.34 | -0.27 | -0.34 | -0.4 | **-0.39** | -0.41 | -0.08 | 0.18 | 0.07 | -0.16 | -0.09 | 0.29 |
|  | Tm | 0.02 | **0.03** | 0.02 | **-0.002** | -0.01 | -0.01 | 0.13 | -0.03 | 0.15 | 0.01 | 0.08 | **-0.14** |
| PCstruct1 | Cc | 0.14 | **0.14** | 0.2 | **0.09** | **0.04** | **0.11** | 0.07 | 0.08 | 0.1 | 0.14 | 0.03 | -0.13 |
|  | Pm | -0.13 | **-0.02** | **-0.17** | -0.09 | -0.05 | -0.14 | 0.09 | **0.4** | 0.07 | **-0.2** | 0.13 | -0.05 |
|  | Tm | 0.2 | 0.17 | 0.19 | **0.25** | 0.25 | 0.23 | **-0.08** | -0.1 | 0.17 | -0.05 | 0.2 | **-0.19** |
| PCstruct2 | Cc | **-0.12** | -0.12 | -0.11 | -0.26 | **-0.26** | -0.26 | -0.04 | 0.2 | 0.18 | 0.001 | -0.1 | 0.11 |
|  | Pm | -0.48** | -0.4 | -0.5** | **-0.54**** | **-0.5**** | **-0.59***** | 0.02 | 0.5** | 0.1 | -0.36 | -0.08 | 0.18 |
|  | Tm | 0.03 | **0.01** | 0.03 | 0.08 | **0.09** | 0.08 | -0.15 | -0.03 | -0.1 | -0.06 | 0.06 | 0.06 |

**Supplementary Table 7** Correlation between song frequency parameters and weather variables.

|  | Species | Air T | Min. air T | Max. air T | Soil T | Min. soil T | Max. soil T | Air pressure | Humidity | Precipitation | Sunshine | Cloudiness | Wind |
| --- | --- | --- | --- | --- | --- | --- | --- | --- | --- | --- | --- | --- | --- |
| max.freq. | Cc | -0.08 | 0.04 | **-0.16** | **-0.05** | 0.02 | **-0.13** | **-0.33** | **-0.003** | 0.1 | -0.31 | 0.02 | **0.17** |
|  | Pm | -0.21 | **-0.25** | -0.2 | **-0.21** | -0.21 | **-0.2** | -0.03 | 0.02 | 0.06 | 0.03 | -0.31 | 0.16 |
|  | Tm | 0.32 | 0.3 | 0.29 | **0.37*** | 0.36* | 0.32 | 0.03 | -0.04 | 0.19 | -0.05 | 0.08 | -0.17 |
| min.freq. | Cc | 0.26 | **0.39** | 0.2 | **0.23** | 0.3 | **0.17** | -0.31 | **-0.05** | 0.11 | **-0.12** | **0.29** | 0.09 |
|  | Pm | 0.03 | 0.06 | **0.01** | **0.1** | 0.08 | 0.1 | **-0.2** | **0.12** | 0.15 | **0.14** | -0.05 | 0.13 |
|  | Tm | -0.16 | -0.15 | -0.15 | -0.18 | -0.18 | -0.17 | 0.09 | 0.1 | -0.14 | -0.05 | -0.02 | -0.02 |
| mean.freq. | Cc | 0.13 | **0.32** | 0.03 | 0.13 | 0.24 | 0.03 | -0.49* | -0.04 | 0.16 | **-0.33** | 0.24 | 0.2 |
|  | Pm | -0.17 | **-0.19** | -0.17 | -0.13 | -0.14 | -0.12 | -0.11 | 0.07 | 0.12 | 0.09 | -0.29 | **0.2** |
|  | Tm | 0.31 | 0.29 | 0.28 | **0.36** | 0.35 | 0.3 | 0.05 | -0.02 | 0.17 | -0.06 | 0.08 | -0.19 |
| max.freq.el | Cc | -0.4 | **-0.45** | **-0.34** | -0.44 | -0.47 | **-0.39** | **0.54**** | **0.2** | -0.23 | -0.1 | -0.16 | -0.2 |
|  | Pm | -0.16 | **-0.22** | -0.12 | **-0.16** | -0.15 | **-0.16** | **0.45*** | 0.22 | -0.06 | -0.15 | -0.03 | -0.27 |
|  | Tm | 0.09 | 0.13 | 0.08 | 0.1 | 0.11 | 0.07 | 0.09 | -0.04 | 0.11 | -0.03 | -0.02 | -0.05 |
| min.freq.el | Cc | 0.03 | 0.03 | 0.08 | 0.001 | **-0.03** | 0.02 | **0.27** | 0.07 | -0.07 | 0.07 | 0.02 | -0.16 |
|  | Pm | -0.19 | -0.19 | **-0.18** | **-0.16** | **-0.16** | -0.14 | 0.37 | 0.19 | -0.13 | -0.13 | **0.17** | -0.23 |
|  | Tm | -0.1 | -0.05 | -0.11 | **-0.13** | -0.12 | -0.15 | 0.16 | 0.09 | 0.02 | **-0.06** | **-0.13** | **0.02** |
| freq.trend.h | Cc | -0.4 | -0.37 | -0.35 | -0.5* | -0.51* | **-0.46** | 0.52* | 0.27 | -0.09 | -0.07 | -0.09 | -0.08 |
|  | Pm | -0.02 | 0.07 | -0.11 | -0.03 | 0.01 | **-0.09** | -0.15 | -0.09 | 0.31 | **0.03** | -0.17 | 0.28 |
|  | Tm | 0.41** | 0.34 | 0.4* | 0.45** | 0.43** | **0.43**** | 0.03 | -0.2 | 0.01 | **0.13** | **-0.05** | -0.15 |
| freq.trend.hAbs | Cc | 0.4 | 0.38 | 0.35 | 0.5* | 0.51* | **0.45** | **-0.51*** | -0.27 | 0.09 | 0.06 | 0.1 | 0.07 |
|  | Pm | **0.17** | **0.06** | 0.19 | 0.19 | 0.17 | 0.2 | -0.05 | **-0.22** | 0.02 | **0.01** | -0.01 | -0.2 |
|  | Tm | 0.4* | 0.35 | 0.38* | **0.44**** | 0.43** | 0.41** | 0.01 | -0.18 | -0.01 | 0.09 | -0.03 | -0.1 |
| freq.trend.l | Cc | -0.36 | -0.38 | -0.3 | **-0.47** | -0.49* | -0.41 | 0.47 | 0.22 | -0.15 | -0.05 | -0.08 | -0.13 |
|  | Pm | -0.001 | 0.07 | -0.08 | -0.02 | 0.01 | **-0.07** | -0.15 | -0.09 | 0.21 | **0.06** | -0.17 | 0.27 |
|  | Tm | 0.36* | 0.25 | 0.38* | 0.4* | **0.37*** | 0.41** | 0.05 | -0.21 | -0.01 | 0.18 | **-0.08** | -0.17 |
| freq.trend.lAbs | Cc | 0.35 | 0.38 | 0.29 | **0.46** | 0.49* | 0.39 | **-0.44** | -0.19 | 0.14 | 0.04 | 0.09 | 0.1 |
|  | Pm | **0.09** | **-0.02** | 0.11 | **0.09** | **0.08** | 0.12 | -0.001 | **-0.21** | -0.17 | **-0.03** | 0.01 | -0.16 |
|  | Tm | 0.36* | 0.25 | 0.37* | 0.41** | **0.38*** | 0.42** | 0.03 | -0.21 | -0.07 | 0.18 | **-0.09** | -0.13 |
| bandwidth | Cc | -0.22 | -0.23 | -0.24 | **-0.19** | -0.19 | **-0.2** | -0.02 | **0.03** | -0.003 | -0.13 | -0.17 | 0.06 |
|  | Pm | -0.2 | **-0.25** | **-0.19** | -0.24 | **-0.24** | **-0.23** | 0.08 | **-0.04** | -0.02 | -0.04 | -0.26 | 0.08 |
|  | Tm | 0.32 | 0.3 | 0.3 | **0.37*** | 0.37* | 0.32 | 0.02 | -0.05 | 0.2 | **-0.04** | 0.08 | -0.16 |
| PCfreq1 | Cc | -0.35 | -0.4 | -0.27 | -0.45 | -0.49* | **-0.37** | **0.58**** | 0.22 | -0.16 | **0.04** | -0.13 | -0.16 |
|  | Pm | 0.16 | **0.23** | 0.14 | 0.16 | **0.17** | **0.14** | -0.13 | **0.01** | 0.02 | 0.04 | 0.2 | 0.04 |
|  | Tm | 0.35 | 0.3 | 0.33 | **0.39*** | 0.38* | 0.36* | 0.04 | -0.13 | 0.09 | 0.05 | -0.01 | -0.15 |
| PCfreq2 | Cc | -0.3 | **-0.34** | -0.29 | **-0.28** | -0.3 | **-0.27** | 0.16 | **0.08** | -0.07 | -0.11 | -0.21 | -0.01 |
|  | Pm | -0.08 | 0.01 | -0.14 | -0.07 | -0.05 | **-0.11** | -0.25 | 0.01 | 0.28 | **0.12** | -0.26 | 0.39 |
|  | Tm | -0.26 | -0.14 | -0.29 | -0.29 | **-0.26** | -0.33 | 0.06 | 0.21 | 0.18 | **-0.21** | 0.05 | 0.08 |
